# A series connection architecture for large-area organic photovoltaic modules with a 7.5% module efficiency

**DOI:** 10.1038/ncomms10279

**Published:** 2016-01-05

**Authors:** Soonil Hong, Hongkyu Kang, Geunjin Kim, Seongyu Lee, Seok Kim, Jong-Hoon Lee, Jinho Lee, Minjin Yi, Junghwan Kim, Hyungcheol Back, Jae-Ryoung Kim, Kwanghee Lee

**Affiliations:** 1 School of Materials Science and Engineering, Gwangju Institute of Science and Technology, Gwangju 61005, Republic of Korea; 2 Heeger Center for Advanced Materials, Gwangju Institute of Science and Technology, Gwangju 61005, Republic of Korea; 3 Research Institute for Solar and Sustainable Energies, Gwangju Institute of Science and Technology, Gwangju 61005, Republic of Korea; 4 Department of Nanobio Materials and Electronics, Gwangju Institute of Science and Technology, Gwangju 61005, Republic of Korea

## Abstract

The fabrication of organic photovoltaic modules via printing techniques has been the greatest challenge for their commercial manufacture. Current module architecture, which is based on a monolithic geometry consisting of serially interconnecting stripe-patterned subcells with finite widths, requires highly sophisticated patterning processes that significantly increase the complexity of printing production lines and cause serious reductions in module efficiency due to so-called aperture loss in series connection regions. Herein we demonstrate an innovative module structure that can simultaneously reduce both patterning processes and aperture loss. By using a charge recombination feature that occurs at contacts between electron- and hole-transport layers, we devise a series connection method that facilitates module fabrication without patterning the charge transport layers. With the successive deposition of component layers using slot-die and doctor-blade printing techniques, we achieve a high module efficiency reaching 7.5% with area of 4.15 cm^2^.

## Abstract (web summary)

The fabrication of organic photovoltaic modules usually relies on patterning techniques which limit their efficiencies. Here, the authors propose a module structure that avoids the patterning steps, and use doctor-blade printing and slot-die coating to fabricate large-area modules reaching 7.5% efficiencies.

## Graphical abstract

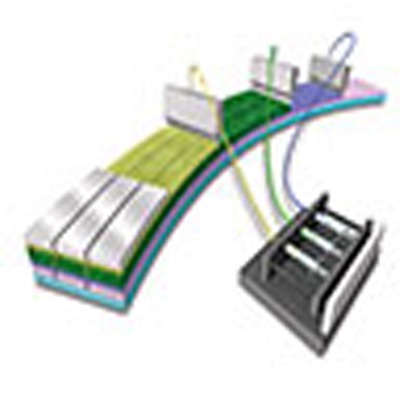

Bulk heterojunction solar cells, which are built on photoactive nanocomposites of electron-donating and electron-withdrawing organic semiconductors, are good candidates to be a ubiquitous renewable energy source that allows for integration with portable and wearable electronic applications^1^,^2^. Moreover, these organic solar cells (OSCs) are considered representative of the research field of printed electronics, because the solution processability of organic semiconductors enables cost-efficient, high-volume/throughput printing production with roll-to-roll manufacturing facility^3^,^4^,^5^,^6^,^7^,^8^. To realize this photovoltaic technology, research has focused mainly on enhancing device performance, extending device lifetime and developing up-scaling techniques for transitioning from small-area laboratory-scale devices to large-area industrial-scale modules^3^,^4^,^5^,^6^,^7^,^8^,^9^,^10^,^11^,^12^,^13^,^14^. Although the impressive progress made in the past two decades has led to considerable improvements in both the efficiency and operational stability of OSCs, the fabrication of large-area printed modules still suffers from significantly reduced power conversion efficiencies (PCEs), amounting to less than half the efficiencies of small-sized laboratory cells.

The current module architecture possesses an inherent weakness with regard to area loss, so-called aperture loss, which is well known to be a major contributor to the drastic performance degradation observed in large-area printed OSC modules^6^,^7^,^8^. The module geometry is based on a monolithic structure composed of several serially interconnecting subcells that are patterned into stripes with sufficiently narrow widths (*W*∼10 mm) to enable the sheet resistance of transparent electrodes to be neglected. However, such monolithic interconnections inevitably produce area loss to ensure contact areas for series connections between subcells. Furthermore, because of the low (millimetre scale) patterning resolutions of current printing techniques, using these techniques to create regularly spaced stripe-patterned subcells worsens unwanted area loss, resulting in very poor module PCEs with low geometric fill factors (FF, ratios between photoactive and total areas). Despite intense research efforts to reduce aperture loss by using laser ablation or metal-filament patterning techniques, only a few methods for realizing high-efficiency printed modules without additional patterning processing have been reported^15^,^16^,^17^.

Here we demonstrate a new module architecture for manufacturing large-area printed OSC modules without the aid of additional and complicated post-patterning processing. By introducing an innovative series connection concept based on the charge recombination characteristic that occurs at the contacts between charge transport layers (CTLs), we design a monolithic interconnection that enables facile and efficient module fabrication without patterning the CTLs and producing the considerable aperture loss. Therefore, through consecutive printing processes using doctor-blade and slot-die machines, we successfully fabricate a large-area module that exhibits a high module PCE of 7.5% with a high geometric FF of 90%.

## Results

### Module architecture and fabrication

**Figure 1:**
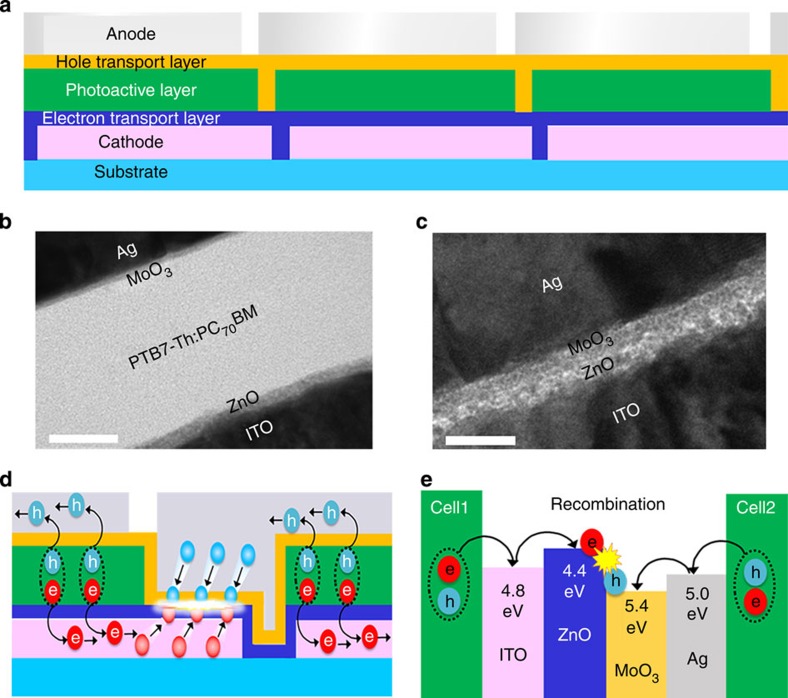
Schematic illustration of the module. (**a**) Conceptual module structure consisting of patternless electron-transport and hole-transport layers and one patterned photoactive layer. (**b**,**c**) Corresponding cross-sectional TEM images of the active area (scale bar, 50 nm) (**b**) and series connection region (scale bar, 25 nm) (**c**). (**d**) A schematic image of charge recombination as it occurs in our module. (**e**) Energy level diagrams of series connection region components.

A schematic illustration of our module architecture is shown in Fig. 1a. The module has three inverted-type subcells consisting of three main component layers sandwiched between an indium tin oxide (ITO) cathode and a silver (Ag) anode. For a photoactive layer, we used a bulk heterojunction composite comprising an electron-donating poly(thieno[3,4-b]thiophene-alt-benzodithiophene) derivative (PTB7-Th) and an electron-accepting [6,6]-phenyl-C_71_-butyric acid methyl ester (PC_70_BM) (ref. 18). To obtain inverted-type subcells, we introduced two CTLs, including sol-gel zinc oxide (ZnO) as an electron-transport layer and molybdenum oxide (MoO_3_) as a hole-transport layer (HTL), between the photoactive layers and their respective electrodes^19^,^20^,^21^. In the module fabrication, the photoactive layer was patterned in stripes onto the patterned ITO cathodes (*W*=13.5 mm) with a slight blank offset (0.5 mm), whereas the two CTLs were deposited on the surfaces of the ITO and photoactive layer in a single-layer form without any stripe patterning. Series connections between adjacent subcells were achieved by forming stripe-patterned Ag anodes (*W*=13.5 mm) with a subtle blank offset (0.5 mm) relative to the patterned photoactive layers. By printing the component layers with a doctor-blade machine for non-patterned single-layer forms and a slot-die machine for stripe patterning, we succeeded in fabricating a monolithic printed module with a high geometric FF of 90% without the use of any post-patterning processing (Supplementary Fig. 1 and Supplementary Note 1).

### Series connection mechanism in the module

The most prominent feature of our module configuration is that the CTLs were not patterned in stripe, in contrast to the conventional modules fabricated with all stripe-patterned component layers (Supplementary Fig. 2). Cross-sectional images taken with a high-resolution transmission electron microscope (TEM) clearly show not only all-component layers (ITO/ZnO/PTB7-Th:PC_70_BM/MoO_3_/Ag) in the active area, but also the CTLs (ZnO/MoO_3_) sandwiched between the ITO and Ag electrodes in a series connection region (SCR), in which the counter electrodes of the adjacent subcells vertically overlap (Fig. 1b,c). Because of the CTLs embedded within the SCRs, our module operation will be quite different from that of typical modules in which the counter electrodes are in direct contact. We can expect the series connection mechanism of our monolithic module to be similar to that of existing multi-junction OSCs; the photogenerated charge carriers (that is, holes and electrons) from neighbouring subcells transport along the counter electrodes and are injected into the CTLs, thereby leading to series connections between subcells and voltage gains via charge recombination at the interface between the CTLs in the SCRs (Fig. 1d,e and Supplementary Fig. 3)^22^,^23^.

### Characteristics of the SCRs

**Figure 2:**
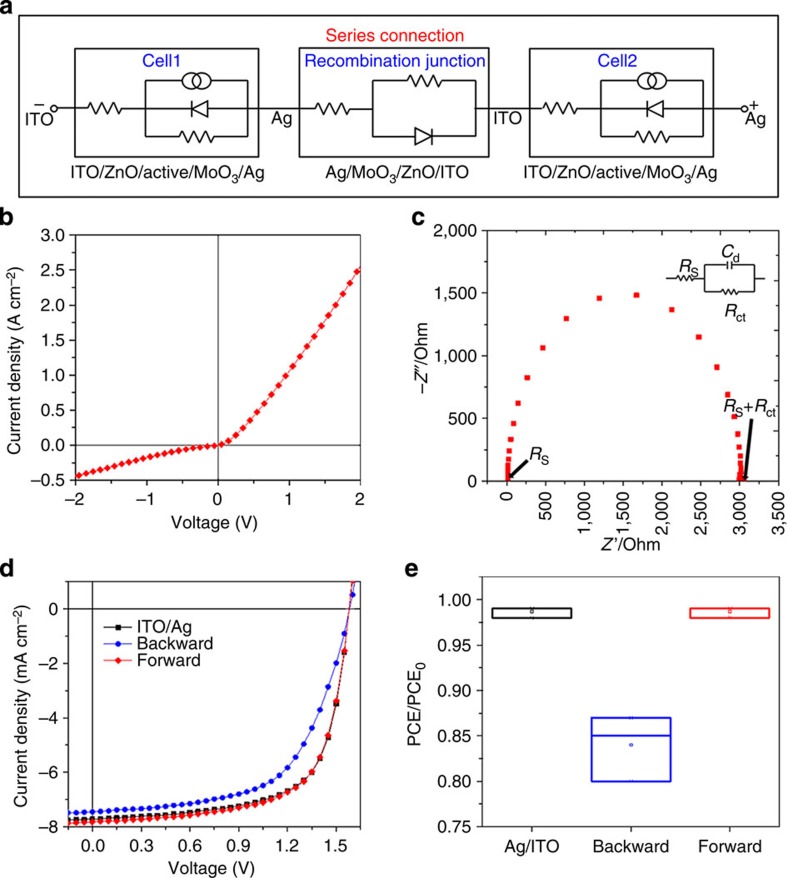
Equivalent circuit of series-connected OSCs with an SCR unit cell. (**a**) The circuit comprised of two OSC unit cells with an SCR unit cell. (**b**) *J–V* characteristic of an SCR unit cell (Ag/MoO_3_/ZnO/ITO) in the dark. (**c**) The Nyquist plot obtained from the EIS analysis of SCR (Ag/MoO_3_/ZnO/ITO). (**d**,**e**) The *J–V* characteristics (**d**) and performance deviations (**e**) of OSCs with an SCR unit cell under AM 1.5G with 100 mW cm^−2^ (PCE_0_ pertains to OSCs without any unit cell, boxes are measured values and rectangular points are average values).

To investigate the impact of the SCRs on module operation, we partitioned the module into a SCR unit cell and two subcells consisting of ITO/ZnO/MoO_3_/Ag and ITO/ZnO/PTB7-TH:PC_70_BM/MoO_3_/Ag, respectively. Using current density–voltage (*J–V*) and electrochemical impedance spectroscopy (EIS) measurements, we formulated the equivalent circuit of the SCR unit cell (Fig. 2a). The *J–V* characteristic of the SCR unit cell shows the rectifying property originating from the electrical junction between the electron-transporting ZnO and hole-transporting MoO_3_ layers (Fig. 2b). Meanwhile, the Nyquist plot obtained via EIS analysis exhibits a low series resistance (*R*_s_) of 12 Ω and a relatively high shunt resistance (*R*_sh_) of 3,000 Ω in the SCR unit cell (Fig. 2c and Supplementary Fig. 4). By combining these results, we can define the SCR unit cell as an electrical component composed of a diode and two resistors. On connecting this SCR unit cell between two subcells, we observed the dependence of device performance on the polarity of the SCR unit cell (Fig. 2d,e). The resulting equivalent circuit reveals that the series connection of the subcells is dominantly affected by the low *R*_s_ of the forward connection and the high *R*_sh_ of the backward connection. Because our module structure connects the forward SCRs with neighbouring subcells, the SCRs are expected to allow loss-free charge recombination of the adjacent subcells in the module operation.

### Optimization of printing processes

**Figure 3:**
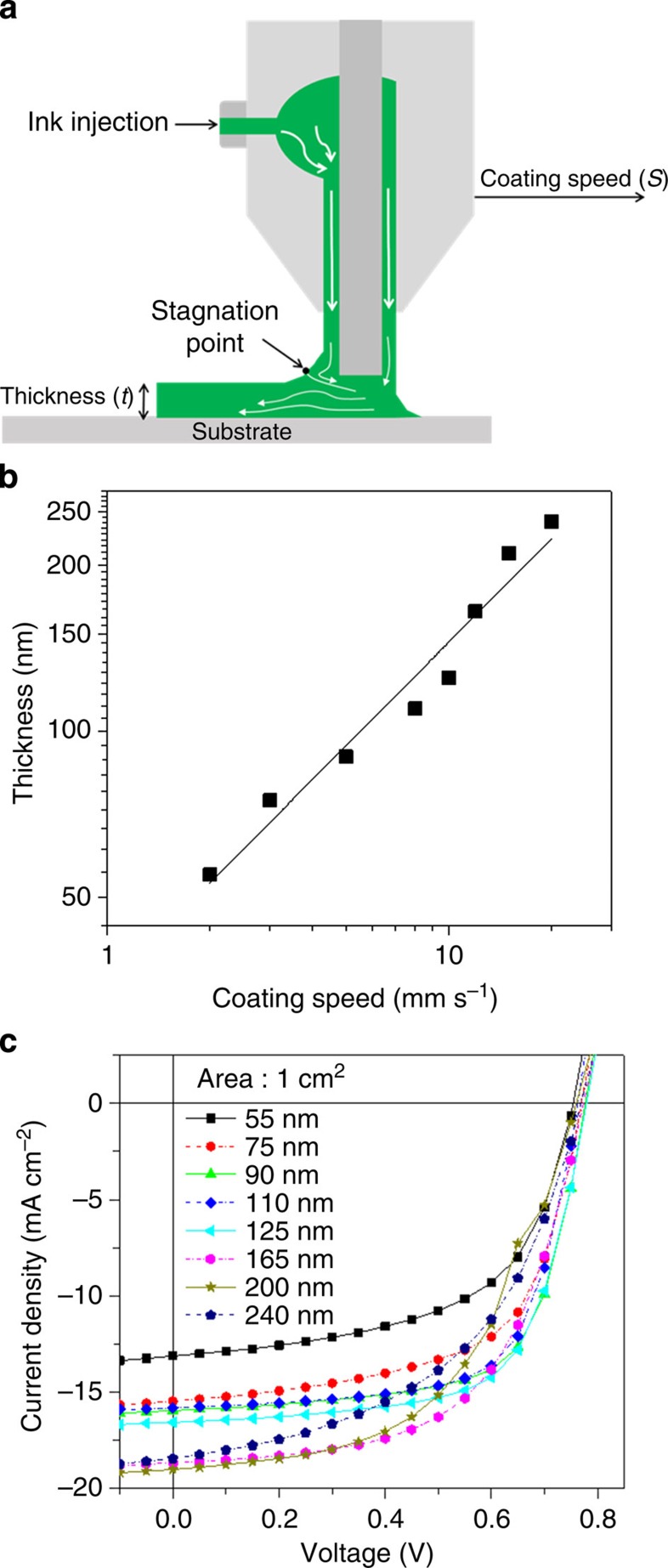
OSCs fabricated using the printing method. (**a**) Schematic of meniscus formation and the streamlines near the stagnation point in the slot-die coating using a positive-shim style mask. (**b**) Thicknesses of PTB7-Th:PC_70_BM films coated via the slot-die coating method using various coating speeds from 2 to 30 mm s^−1^ (log scale). (**c**) *J–V* characteristics of OSCs fabricated using the slot-die coating method at various thicknesses.

To fabricate our printed module, we employed two kinds of printing techniques using doctor-blade and slot-die machines (Supplementary Fig. 5). Both printing machines have similar control factors, which depend on the viscosity of the solution, the amount of feeding solution, the coating speed and the substrate temperature. By delicately adjusting these parameters, we achieved high-quality printed films with smooth and uniform film morphologies (Supplementary Fig. 6). In particular, we designed a slot-die coating head with a positive shim mask to create a meniscus guide (Supplementary Fig. 7); the photoactive PTB7-Th:PC_70_BM solution was ejected through a narrow slot and followed the shim mask pattern, thereby forming a meniscus between the mask and substrate via capillary action (Fig. 3a). To simply control the film thickness (*t*), we changed the coating speed (*S*) while fixing other coating parameters. As shown in Fig. 3b, the thicknesses of PTB7-Th:PC_70_BM films follow power law equation of *t≈S*^0.62^, which has been demonstrated in meniscus coating methods using low-viscosity organic solutions^24^,^25^. After optimizing the film thicknesses, we obtained a high-quality printed photoactive layer with a thickness of ∼125 nm that exhibited an optimal PCE of 8.5% (Fig. 3c, Supplementary Fig. 8 and Supplementary Table 1).

### Performance of large-area printed OSC modules

**Figure 4:**
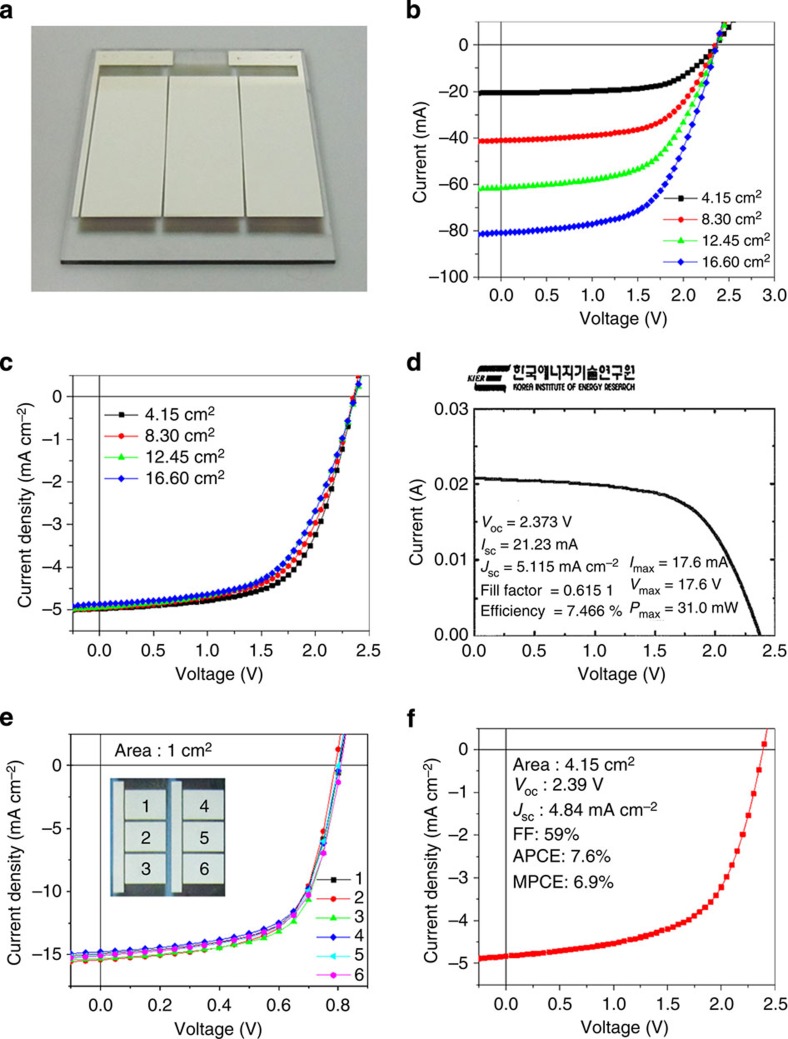
OSC modules fabricated using the printing method. (**a**) A photograph image of our module (size of 60 × 44.5 mm). (**b**,**c**) *I–V* (**b**) and *J–V* (**c**) characteristics of OSC modules of various sizes depending on module lengths from 1 to 4 cm. (**d**) Korea Institute of Energy Research-certified *J–V* characteristics of our OSC module. (**e**,**f**) *J–V* characteristics of single OSCs in different positions (inset: photograph image of printed OSCs) (**e**) and OSC module (**f**) by using the printed MoO_3_ layer. APCE is the PCE of the module in active area and MPCE is the PCE of the module in total area.

Figure 4a displays a photograph of the complete large-area printed module with optimized film thicknesses. The current–voltage (*I*–*V*) and current density–voltage (*J–V*) characteristics of the module, which are measured via a large-scale calibrated solar simulator under standard illumination conditions, are shown in Fig. 4b,c and Table 1. The best performance of the new module (4.15 cm^2^) yielded a remarkable PCE of 8.1% with an open-circuit voltage (*V*_oc_) of 2.36 V, a short-circuit current density (*J*_sc_) of 5.53 mA cm^−2^, and a FF of 62%. The *V*_oc_ (2.36 V) of the module is nearly three times larger than the *V*_oc_ (0.79 V) of the small-area reference; the *J*_sc_ (5.53 mA cm^−2^) of the module is exactly one-third of that (16.6 mA cm^−2^) of the reference; and the FF (62%) of the module is almost comparable to that of the reference (66%). Considering its geometric FF of 90%, the module exhibits a high module PCE of 7.3%. One of the modules exhibiting the best PCE was sent to the Korea Institute of Energy Research and was returned to our laboratory with a certificated module PCE of ∼7.5%, as shown in Fig. 4d. To the best of our knowledge, this PCE is the highest value in printed solar modules to date in scientific literature. These outstanding results indicate that the subcells were perfectly interconnected via effective charge recombination at the interfaces between the CTLs in the SCRs. Although increasing the length (size) of the module causes a slight decrease in the FF and *J*_sc_ values due to a few concomitant defect sites (for example, pinholes and fine dusts) within the printed films, we can overcome this problem through defect-free printing process in clean rooms, thereby enlarging the module size without suffering serious performance loss^6^,^26^. In addition, we introduced a solution-processed MoO_3_ layer for the module fabrication^27^. By printing the MoO_3_ layer, we obtained reasonable average efficiencies of 7.7% for large-area single cells and 6.9% for module, thereby demonstrating that our module can operate well even when we used all-printed CTLs (Fig. 4e,f and Supplementary Table 2).

## Discussion

Our work demonstrates a new scientific perspective, in that we have designed a simple and efficient module structure by developing a novel series connection method and a remarkable technical advance towards manufacturing printed modules for next-generation photovoltaic systems. By using a charge recombination feature arising from the electrical junctions between CTLs, we succeed in fabricating a monolithic module without the use of additional complex patterning processes. This monolithic module has achieved a certificated module PCE of 7.5% with a high geometric FF of 90%. We expect that this new approach presents a simple and useful means for transitioning from small-area laboratory-scale OSCs to large-area industrial-scale OSC modules.

## Methods

### Material preparation

The ZnO precursor was prepared by dissolving zinc acetate dihydrate (Zn(CH_3_COO)_2_·2H_2_O, Aldrich, 99.9%, 1 g) and ethanolamine (NH_2_CH_2_CH_2_OH, Aldrich, 99.5%, 0.5 g) in isopropyl alcohol (CH_3_OCH_2_CH_2_OH, Aldrich, 99.8%, 50 g) via stirring for 24 h. The PTB7-Th:PC_70_BM solution was prepared by blending PTB7-Th (1-material) and PC_70_BM (Nano-C) at a ratio of 1:2 in chlorobenzene solvent with 1,8-diiodooctane additive (3% by volume) to obtain a total concentration of 12 mg ml^−1^. The MoO_3_ solution was prepared by dissolving bis(acetylacetonato) dioxomolybdenum (Sigma Aldrich) in a cosolvent of methanol and 1-butanol.

### Single-cell fabrication

ITO/glass substrates were cleaned with detergent, after which they were sequentially washed via ultrasonic treatment in de-ionized water, acetone and IPA. The ZnO solution was coated onto the ITO/glass substrate using a doctor-blade coater (Coatmaster 509 MC, Erichsen in Germany) at 40 °C, then annealed at 150 °C for 20 min in air. The PTB7-Th:PC_70_BM composite solution was coated on top of the ZnO layer in air using a slot-die coating method (Slot-die coater, iPen in South Korea) at room temperature. The pumping rate for coating PTB7-Th:PC_70_BM solution was 0.1 ml min^−1^ when using a 50-μm-thick mask, and the film thickness was controlled by the coating speed of the slot-die header. To complete the single-cell device fabrication, MoO_3_ (as a HTL) and Ag (as a top electrode) were deposited sequentially by thermal evaporation in a vacuum with a pressure of 10^−6^ torr.

### Module fabrication

The ITO/glass preparation and ZnO coating process is the same as that described for single-cell fabrication. Before the module fabrication, the patterned ITO glass substrate (the stripe width of 13.5 mm and the gap of 0.5 mm) was prepared by wet-etching processing using a typical acid etchant. For the patterned PTB7-Th:PC_70_BM coating process, we used a slot-die coating machine with a 50-μm-thick three-stripe mask. The coating speed and pumping rate used to coat the PTB7-Th:PC_70_BM solution are 10 mm s^−1^ and 0.4 ml min^−1^, respectively, and the optimized thickness of PTB7-Th:PC_70_BM is ∼125 nm. The MoO_3_ (as a HTL) was deposited onto the patterned photoactive layer with no patterned mask by using thermal evaporation in a vacuum with a pressure of 10^−6^ torr. The solution-processed MoO_3_ layer was deposited on the photoactive layer by using a doctor-blade coating machine. Module-device fabrication was completed by thermal evaporation of the Ag metal top electrode (200 nm thickness) in a vacuum with a pressure of 10^−6^ torr. In contrast to MoO_3_ deposition, Ag was deposited via a patterned mask, which is used to obtain a series connection in the module.

### Characterization and analysis

The current–voltage (*I–V*) characteristics were recorded using an Iviumsoft apparatus with simulated AM 1.5 illumination (100 mW cm^−2^) via a solar simulator (Abet Technologies Sun 3000) in normal air conditions. The thicknesses of the coated films were measured using a thickness profile metre (Surfcorder ET 3000, Kosaka Laboratory, Ltd.). Cross-sectional TEM samples of the printed OSC module were prepared using a dual beam-focused ion beam (Helios NanoLabTM). The TEM images were obtained using field emission TEM (FEI TecnaiTM G2 F30 Super-Twin) operated at 200 kV. The topographies of surface images were characterized using atomic force spectroscopy.

## Additional information

**How to cite this article:** Hong, S. *et al.* A series connection architecture for large-area organic photovoltaic modules with a 7.5% module efficiency. *Nat. Commun.* 7:10279 doi: 10.1038/ncomms10279 (2016).

## Supplementary Material

Supplementary Information Supplementary Figures 1-8, Supplementary Tables 1-2 and Supplementary Note 1

## Figures and Tables

**Table 1:** Performance parameters of the OSC modules with increasing area.

**Area (cm**^2^**)**	***V***_**OC**_ **(V)**	***I***_**SC**_ **(mA)**	***J***_**SC**_ **(mA cm**^−2^**)**	**FF (%)**	**APCE (%)**	**MPCE (%)**
4.15	2.36	20.7	4.98	62	8.1	7.3
8.30	2.36	41.1	4.95	60	7.7	7.0
12.45	2.38	61.6	4.94	57	7.4	6.7
16.60	2.37	80.9	4.87	58	7.4	6.7

APCE, the PCE of the module in active area; MPCE, the PCE of the module in total area.
