# Supplementary material for: A series connection architecture for large-area organic photovoltaic modules with a 7.5% module efficiency
Source: Nat Commun. 2016 Jan 5;7:10279. doi: 10.1038/ncomms10279 (PMC4728442; doi:10.1038/ncomms10279)
Supplement: Supplementary Information — Supplementary Figures 1-8, Supplementary Tables 1-2 and Supplementary Note 1 [file ncomms10279-s1.pdf]

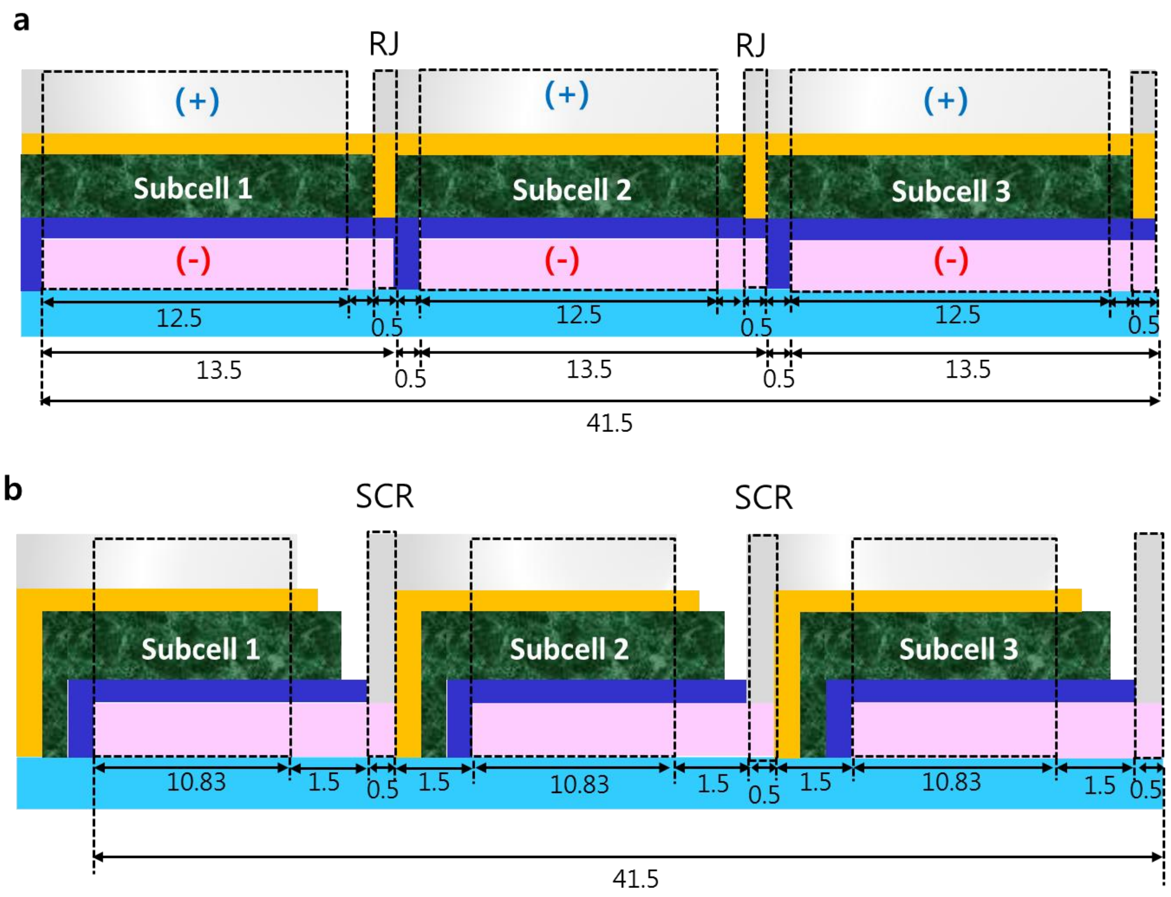

Supplementary Figure 1 | Components, geometrical fill factor of our module (a) and conventional module (b). Our module consists of three subcells and two SCRs, with patterned photo active layer and electrodes (13.5 mm wide), blank offsets (1.0 mm wide) between electrodes, a photoactive region (12.5 mm wide) and SCRs (0.5mm wide) between the counter electrodes of the adjacent subcells. The calculated geometric fill factor (photoactive area/total area) is 90%. In contrast, the conventional module results in the low geometric fill factor of 78%, considering that each layer is shifted with respect to the underlying layers (0.5 mm).

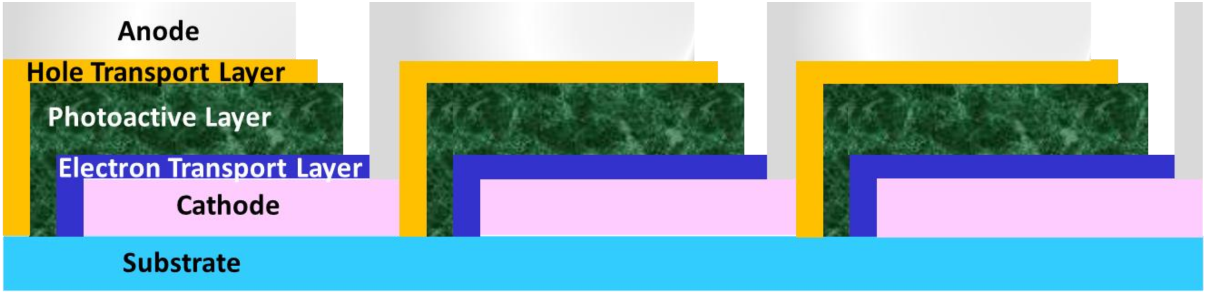

Supplementary Figure 2 | Conventional module structure. The conventional modules cause significant aperture loss due to the patterned coating process.

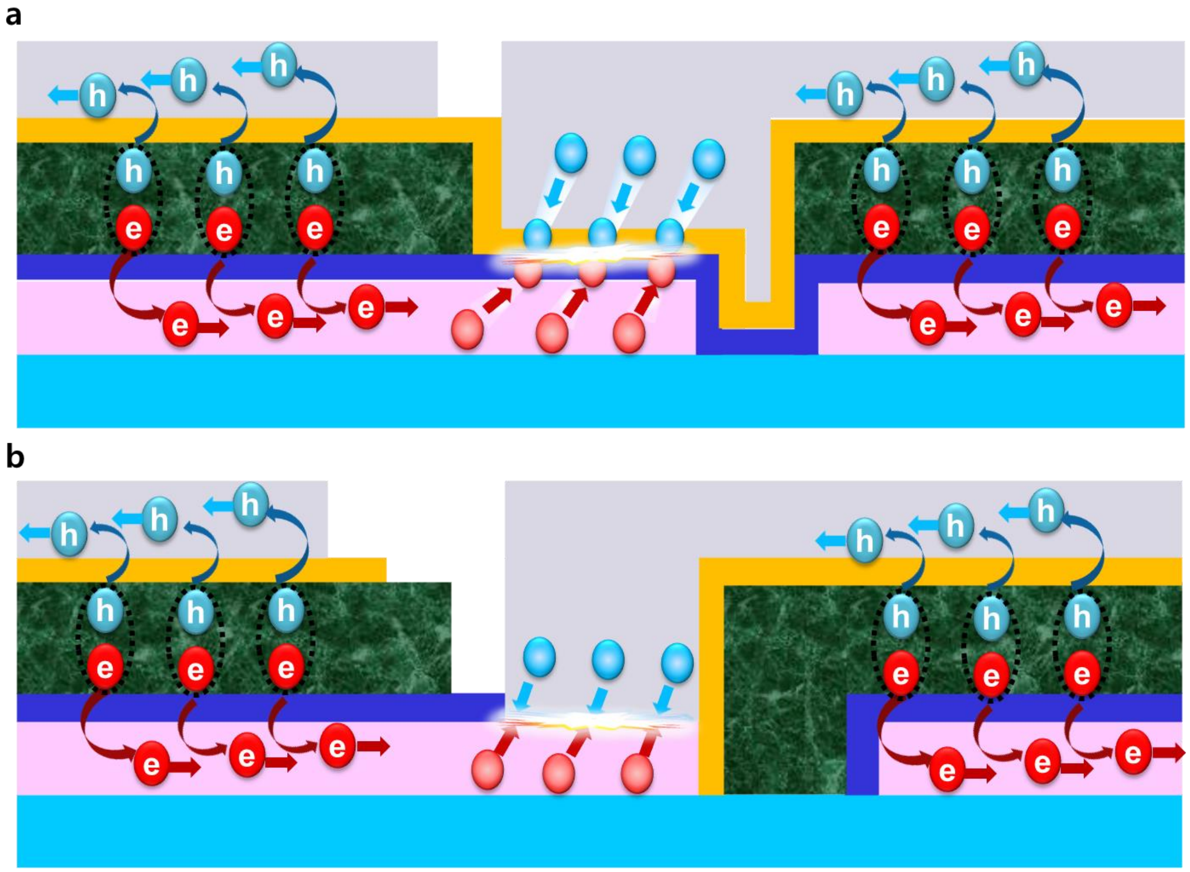

Supplementary Figure 3 | Working mechanisms for our module (a) and conventional module (b). In our module, photogenerated electrons and holes from each sub-cell transport along the counter electrodes and are injected into the CTLs of the SCRs, and recombine together at the interface between the CTLs. In contrast, the conventional module results in charge recombination at the interface between the counter electrodes of the SCRs.

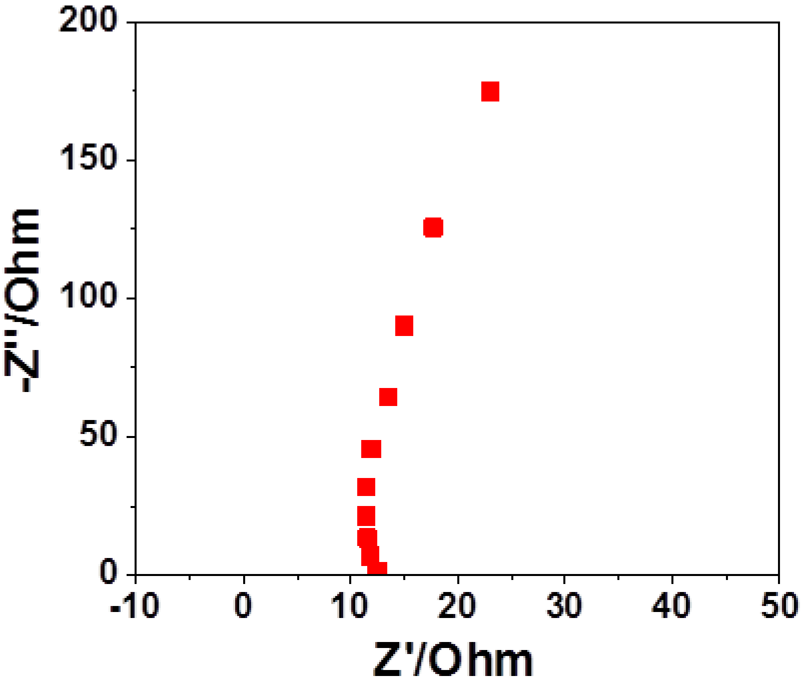

Supplementary Figure 4 | Enlarged image of the Nyquist plot obtained from the EIS analysis of SCR (Ag/MoO3/ZnO/ITO).

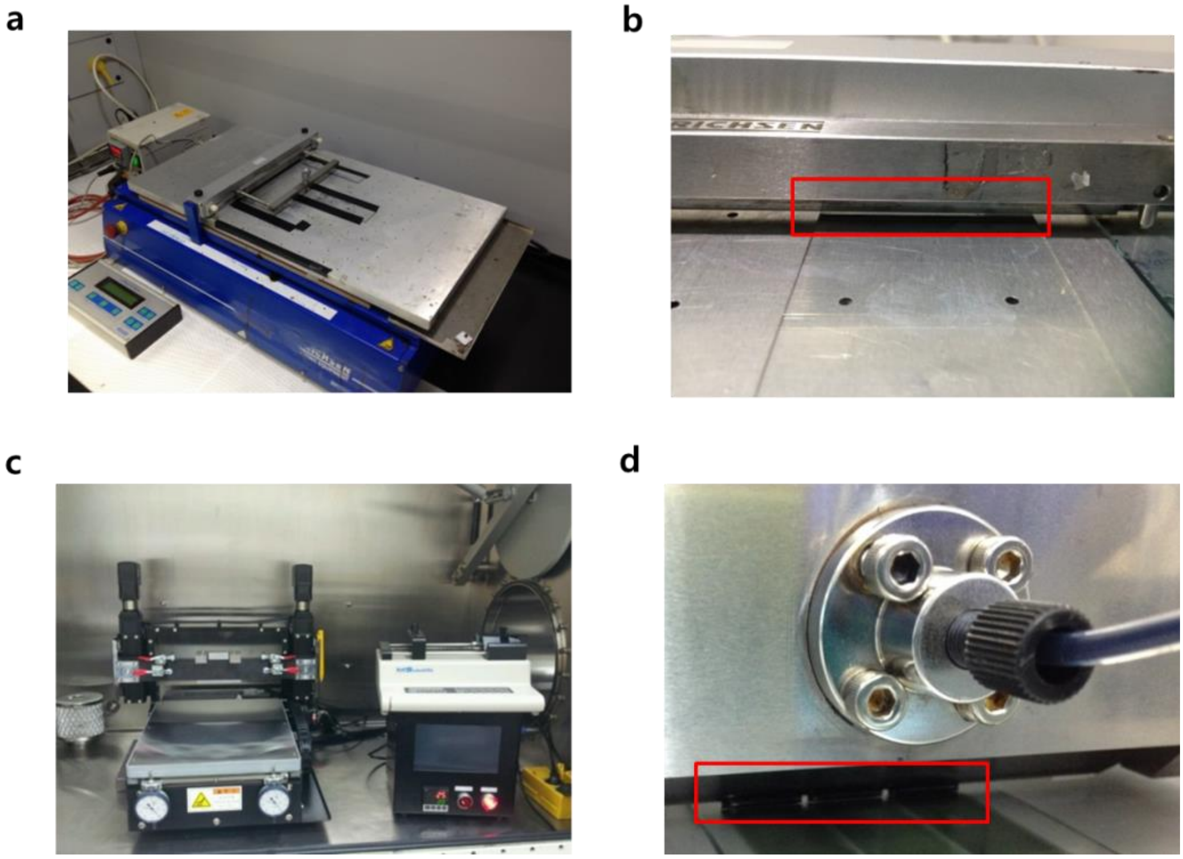

Supplementary Figure 5 | Photographs of doctor blade coater (a) and coating of the ZnO material onto ITO/glass substrate (b), and slot-die coater (c) and coating of the PTB7-Th:$PC_{70}BM$ material onto ZnO films (d).

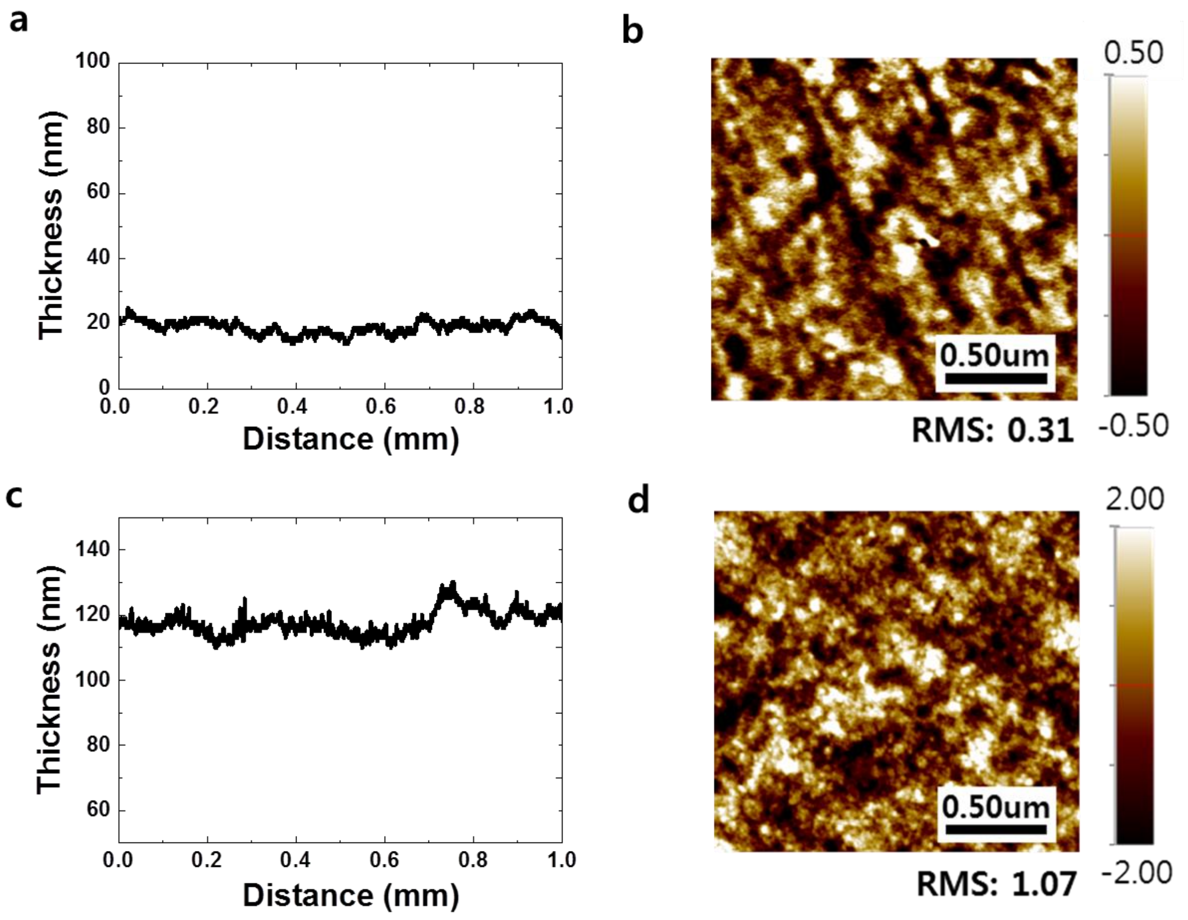

Supplementary Figure 6 | Thickness profiler and AFM topography (2 um × 2 um) images of printed ZnO films (a,b) and PTB7-Th:$PC_{70}BM$ films (c,d).

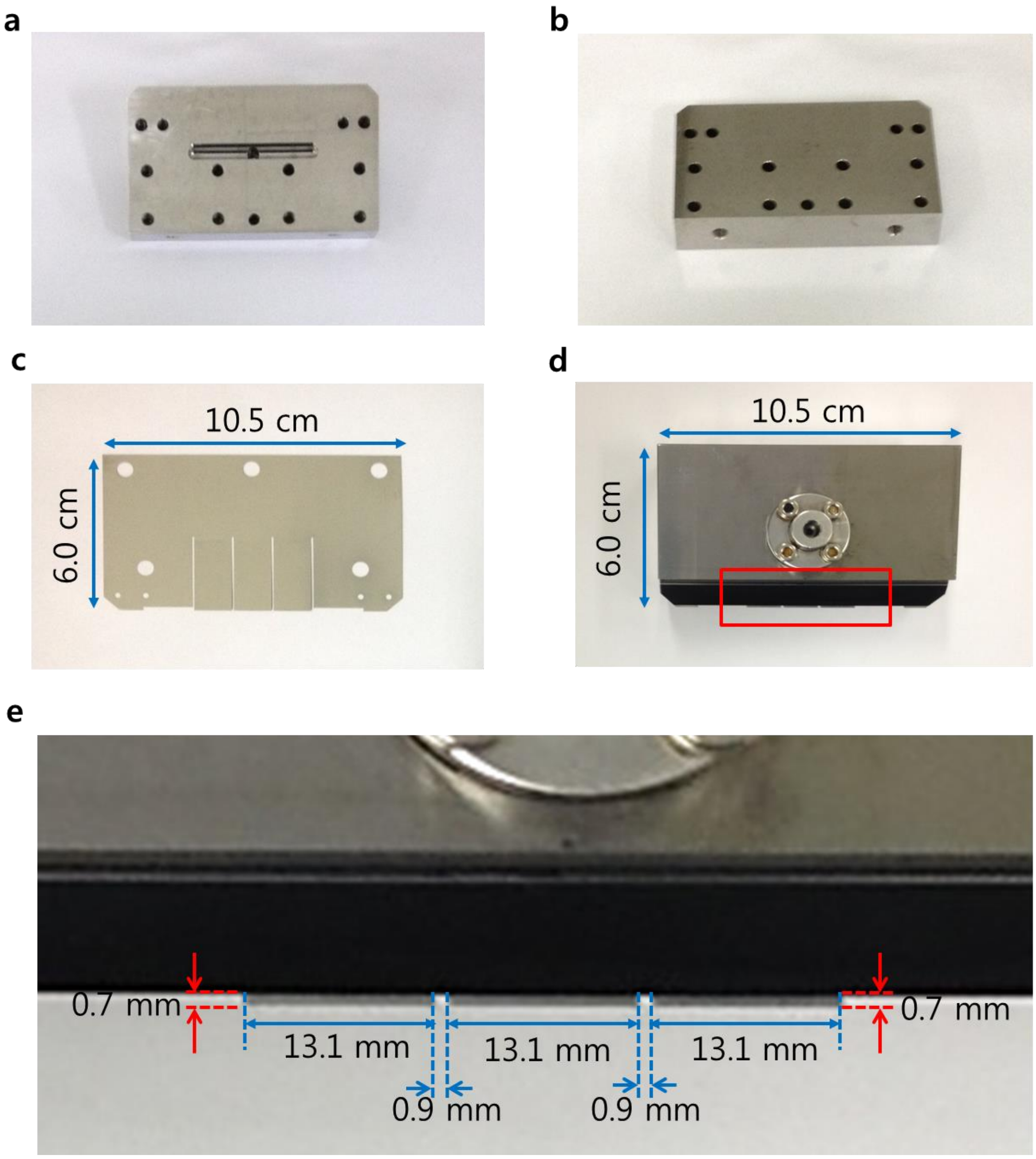

Supplementary Figure 7 | The bottom (a) and top (b) plate of slot-die head, a positive shim style mask (c), assembly of slot-die head with positive shim style mask (d), and a close-up photograph of the slot-die head (e).

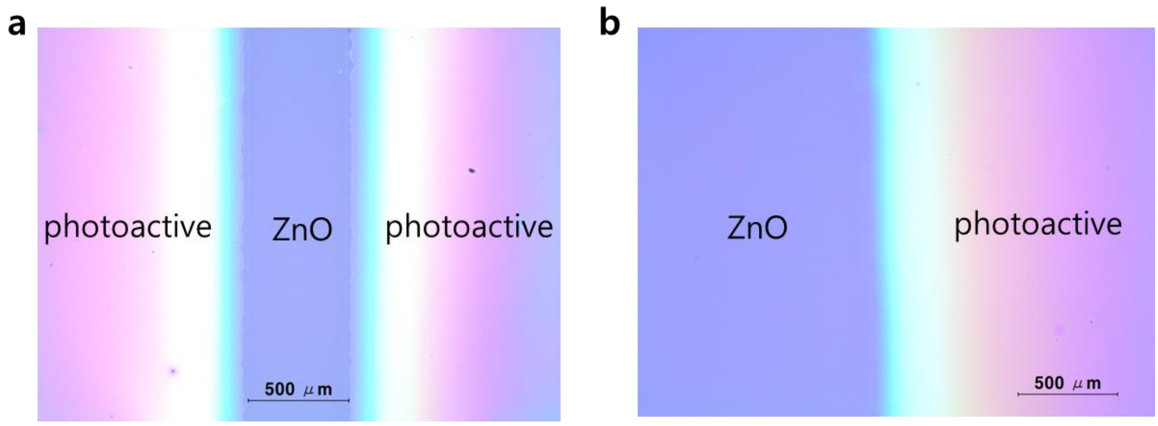

Supplementary Figure 8 | Optical microscope images of the gap between the printed photoactive layers (left) and the edge of the photoactive layer (right).

Supplementary Table 1 | Performance parameters of the printed OSCs with increasing the thickness of the photoactive layer.

Thickness (nm)	V_{OC} (V)	J_{SC} (mA cm^{-2})	FF (%)	PCE (%)
55	0.76	13.9	57	6.0
75	0.77	15.5	61	7.3
90	0.78	15.9	67	8.3
110	0.76	15.8	68	8.2
125	0.78	16.6	66	8.5
165	0.77	18.7	58	8.4
200	0.76	19.0	53	7.6
240	0.77	18.4	49	7.0

Supplementary Table 2 | Performance parameters of OSCs using the printed MoO_3 layer with different positions.

Cell Position	V_{OC} (V)	J_{SC} (mA cm^{-2})	FF (%)	PCE (%)
1	0.80	14.9	63	7.6
2	0.79	15.4	64	7.7
3	0.80	15.3	66	8.0
4	0.80	14.8	63	7.5
5	0.80	15.0	64	7.7
6	0.81	15.1	63	7.7
Average	0.80	15.1	64	7.7

Supplementary Note 1

We calculate geometrical fill factor of our module following below equation.

$$Geometrical\ Fill\ Factor\ = \frac{Photoactive\ Area}{Total\ Area}$$

$$= \frac{12.5 \times 3}{13.5 \times 3 + 2 \times 0.5} \times 100\ = \frac{37.5}{41.5} \times 100 = 90\%$$

We calculate geometrical fill factor of conventional module following below equation.

$$Geometrical\ Fill\ Factor\ = \frac{Photoactive\ Area}{Total\ Area}$$

$$= \frac{10.83 \times 3}{13.5 \times 3 + 2 \times 0.5} \times 100\ = \frac{32.5}{41.5} \times 100 = 78\%$$
